# Supplementary material for: Cause-Specific Stillbirth and Neonatal Death According to Prepregnancy Obesity and Early Gestational Weight Gain: A Study in the Danish National Birth Cohort
Source: Nutrients. 2021 May 15;13(5):1676. doi: 10.3390/nu13051676 (PMC8156544; doi:10.3390/nu13051676)
Supplement: Supplementary file 1 [file nutrients-13-01676-s001.zip › nutrients-1202064-supplementary.pdf]

Supplementary Table 1.
Classification system[a] for primary cause of death - 302 singleton late stillbirths[b] and 255 singleton neonatal deaths[b] in 100,413 pregnancies included in The Danish National Birth Cohort.

Categories	Criteria	Stillbirth/neonatal death	Number of deaths
**Congenital malformations**	Chromosome abnormalities<br>Neural tube defects<br>Inborn errors of metabolism<br>Heart defects<br>Renal abnormalities<br>Other abnormalities	Stillbirth and neonatal death	134
**Unexplained intrauterine death**	Unexplained death among fetuses that were normal weight for gestational age.	Only stillbirth	84
**Placental dysfunction [c]**	Intrauterine growth retardation (IUGR)<br>Infarction of placenta without IUGR<br>Placental abruption<br>Placenta previa	Stillbirth and neonatal death	106
**Umbilical cord complications [d]**	Fetal entanglement<br>Cord knot<br>Perforation of the umbilical cord<br>Vasa previa<br>Thrombosis	Stillbirth and neonatal death	43
**Antepartum bleeding [e]**	Excess bleeding of unknown origin	Stillbirth and neonatal death	< 3
**Maternal conditions**	Preeclampsia<br>Hypertension<br>Diabetes mellitus<br>Rhesus isoimmunization<br>Other maternal conditions	Stillbirth and neonatal death	22
**Intrapartum events**	Birth trauma<br>Irregular fetal presentation<br>Rupture of the uterus<br>Aspiration of meconium<br>Intrapartum asphyxia, unknown cause	Stillbirth and neonatal death	55
**Preterm birth**	Immature respiratory system<br>Hyaline membrane disease (HMD)<br>HMD with intraventricular bleeding<br>HMD with infection<br>Intracranial bleeding including<br>Intraventricular bleeding	Only neonatal death	43
**Infections**	Necrotizing enterocolitis (NEC)<br>Ante-, intra- and postpartum infection	Stillbirth and neonatal death	35
**Other specific conditions**	Specific conditions including sudden infant death.	Stillbirth and neonatal death	23
**Unclassifiable**	death from any cause not classifiable to any of the other categories or lack of information	Stillbirth and neonatal death	11

[a] A modified version of the classification suggested by Andersen KV et al in [Classification of perinatal and neonatal deaths. Fetal, obstetrical and neonatal causes]. Ugeskr Laeger 1991. 153;1494-1497. [b] Late stillbirth defined as stillbirths after 28 completed weeks of gestation, neonatal death as deaths within the first 28 days of life. [c] This category has been modified from the original system to include only placental dysfunction and both stillbirth and neonatal death. [d] This category was added to the original classification system. It was originally a subcategory in a category named 'fetal-placental dysfunction'. [e] This category has been modified from the original system: The subcategories 'Placental abruption' and 'Placenta previa' has been moved to the category 'Placental dysfunction'.
